# *Announcement:* Community Preventive Services Task Force Findings for Mobile Phone Applications Used Within Health Care Systems for Self-Management of Type 1 and Type 2 Diabetes

**DOI:** 10.15585/mmwr.mm6649a7

**Published:** 2017-12-15

The Community Preventive Services Task Force (CPSTF) recommends mobile phone applications used within health care systems for self-management of type 2 diabetes. “Diabetes Management: Mobile Phone Applications Used Within Healthcare Systems for Type 2 Diabetes Self-Management” is available at https://www.thecommunityguide.org/findings/diabetes-management-mobile-phone-applications-used-within-healthcare-systems-type-2.

The CPSTF finds insufficient evidence for the intervention approach when used with patients who have type 1 diabetes. “Diabetes Management: Mobile Phone Applications Used Within Healthcare Systems for Type 1 Diabetes Self-Management” is available at https://www.thecommunityguide.org/findings/diabetes-management-mobile-phone-applications-used-within-healthcare-systems-type-1.

Established in 1996 by the U.S. Department of Health and Human Services, the CPSTF is an independent, nonfederal panel of public health and prevention experts whose members are appointed by the director of CDC. The CPSTF provides information for a wide range of persons who make decisions about programs, services, and other interventions to improve population health. Although CDC provides administrative, scientific, and technical support for the CPSTF, the recommendations developed are those of the task force and do not undergo review or approval by CDC.

